# Efficient neural codes can lead to spurious synchronization

**DOI:** 10.3389/fncom.2013.00125

**Published:** 2013-09-10

**Authors:** Massimiliano Zanin, David Papo

**Affiliations:** ^1^Departamento de Engenharia Electrotécnica, Faculdade de Ciências e Tecnologia, Universidade Nova de LisboaLisboa, Portugal; ^2^Innaxis Foundation and Research InstituteMadrid, Spain; ^3^Center for Biomedical Technology, Technical University of MadridMadrid, Spain

**Keywords:** synchronization, neural models, boolean code, EEG/MEG, stimuli

Experimental and computational evidence shows that cognitive function requires an optimal balance between global integrative and local functionally specialized processes (Tononi et al., 1998). This balance can be described in terms of transient short-lived episodes of synchronized activity between different parts of the brain (Friston, 2000; Breakspear, 2002). Synchronization over multiple frequency bands is thought to subserve fundamental operations of cortical computation (Varela et al., 2001; Fries, 2009), and to be one of the mechanisms mediating the large-scale coordination of scattered functionally specialized brain regions. For instance, transient synchronization of neuronal oscillatory activity in the 30–80 Hz range has been proposed to act as an integrative mechanism, binding together spatially distributed neural populations in parallel networks during sensory perception and information processing (Singer, 1995; Miltner et al., 1999; Rodriguez et al., 1999). More generally, synchrony may subserve an integrative function in cognitive functions as diverse as motor planning, working or associative memory, or emotional regulation (Varela, 1995).

Over the past 15 years, cognitive neuroscientists have tried to capture and quantify neural synchronies across distant brain regions both during spontaneous brain activity and in association with the execution of a wide range of cognitive tasks, using neuroimaging techniques such as functional resonance imaging, electro- or magneto-encephalography. Theoretical advances in various fields including non-linear dynamical systems theory have allowed the study of various types of synchronization from time series (Pereda et al., 2005), and to address important issues such as determining whether observed couplings do not reflect a mere correlation between activities recorded at two different brain regions but rather a causal relationship (Granger, 1969) whereby a brain region would cause the activity of the other one.

However, not all measured synchrony may in fact represent neurophysiologically and cognitively relevant computations: various confounding effects may mislead into identifying functional connectivity, defined as the temporal correlations between spatially remote neurophysiological events, with effective connectivity, i.e., the influence one neuronal system exerts over another (Friston, 1994). For instance, measured synchrony may stem from common thalamo-cortical afferents or neuromodulatory input from ascending neurotransmitter systems, or may be the visible part of indirect effective connectivity. Other technique-specific artifactual sources of synchrony, for instance induced by volume conduction, are also well-known to cognitive neuroscientists (Stam et al., 2007).

Here, we address a further (extra-cranial) confounding source: the appearance of simultaneous, yet uncorrelated stimuli. We show how the activity of two groups of binary neurons, whose output code is optimized to represent rare events with short codes, can exhibit a synchronization when such rare events appear, even in the absence of shared information or common computational activities.

## 1. The model

We suppose that a neuron codifies an external stimulus with a set of spikes, to transmit information about the event to other regions of the neural system. For the sake of simplicity, let's also suppose that all stimuli are drawn from a finite set of events E = {*e*_1_,…, *e*_N_}, *N* being the total number of events. Each event *i* is characterized by two strongly related features: the frequency of appearance *f*_*i*_ and the importance factor *m*_*i*_. Clearly, rare events are also the most important ones. For instance, the image of a group of trees is quite common for an animal, and should not attract his attention. On the other hand, a predator appearing behind such trees is far less frequent, and the importance of a fast response to the event, high. Therefore, for each event *i*, the relation *m*_*i*_ = 1/*f*_*i*_ is defined.

Each neuron optimizes its code to represent such an environment, i.e., it assigns a symbol *s*_*i*_ drawn from an alphabet 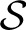 to each input event *i*. As the neuron natural language is composed of spikes, each symbol *s*_*i*_ is defined as a sequence of spikes and silences; this is represented by a sequence of 0's and 1's, of arbitrary length, forming a Boolean code. In other words, and from an information science perspective, each symbol *s*_*i*_ is a number in its Boolean representation.

In the creation of the code, the neurons use all their available knowledge concerning their environment, given by *f*_*i*_ and *m*_*i*_, trying to fulfilling two conditions. First, the cost associated with the transmission of information should be minimized, thus as few spikes as possible should be generated; this favors large symbols with few 1's and a large proportion of 0's. This condition is energy saving, but increases the neuron's response time. Therefore, a second condition ensures that the neuron minimizes symbol length, particularly those associated with events or items of great importance, i.e., with low *f*_*i*_ and high *m*_*i*_.

A *cost* given by:

(1) $$C=\sum_{i}\left[\alpha \frac{b_{i}f_{i}}{l_{i}}+\left(1-\alpha \right)l_{i}m_{i}\right]$$

accounts for the trade-off between these conditions is associated to each code, and minimized by the neuron in a training phase representing a natural selection process. The contribution of each symbol *i* to the total is given by two terms—see Equation 1. The first, involving the number of spikes in the symbol (*b*_*i*_), its expected frequency of appearance (*f*_*i*_) and its length (*l*_*i*_), expresses the probability of having the neuron spiking, at a given time, and thus the expected energetic cost of the code. The second term penalizes the appearance of long symbols codifying important messages. Finally, the parameter α defines the balance between both contributions to the total cost: for α ≈ 0 (α ≈ 1) the total cost is dominated by the length of important symbols (by the energetic cost).

Two additional requirements are added. First, for different events no to be confused, all symbols should be different, i.e., *s*_*i*_ ≠ *s*_*j*_. Second, all symbols should start with a spike (a 1) and have at least one zero, in order to be recognizable and to avoid codes composed only of silences or spikes.

Due to the computational cost of optimizing such codes when multiple events are considered, the process is performed by means of a *greedy algorithm* Cormen et al. (2001), that is, by starting with an empty set, and adding one symbol at the time, making the locally optimal choice at each iteration.

## 2. Results

We now explore how a spurious synchronization between different neurons (or groups of them) can be achieved even in the absence of any information transfer.

Neurons are supposed to work independently, that is, they receive independent inputs from the environment and create their optimal code to process and transmit such information. For instance, two groups of neurons may receive two different and uncorrelated stimuli, corresponding to the image of a predator and the sound of a thunder.

Following this idea, a large number of neurons are modeled and their codes created. Each neuron has its independent set of stimuli, half of them highly probable (and therefore, less important), and half of them with low probability of appearance.

Using this information, all codes are generated, and a time series for each neuron is created, by presenting sequences of stimuli at random, and recording the neuron's corresponding activity. Time series are divided into two parts of equal length. During the first half, neurons are stimulated by high-probability events; the opposite occurs during the second half. Following the previous example, we suppose that the organism is resting quietly at the beginning, and then spots a predator and hears a thunder. Furthermore, we suppose that neurons do not respond with the same velocity to the external stimuli: each neuron receives its inputs with a delay drawn from a uniform distribution defined between 0 and 400 time steps.

**Figure 1:**
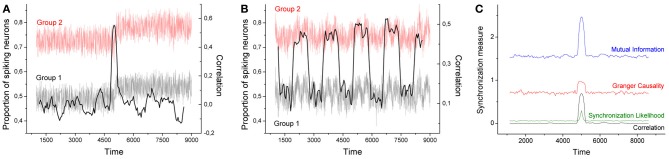
**(**A,B**)** Time series of the proportion of spiking neurons generated by two groups of 500 neurons (gray and red lines). In panel **A** (panel **B**), the probability of finding rare events is changed at time 5000 (is continuously changed). Time series of group 2 is represented with an offset of 0.25. The black solid line represents the evolution of the Pearson's correlation coefficient between both groups, calculated with a sliding window of size 400. **(C)** Average values for the four synchronization metrics, using the same event sets of panel **(A)**. All neural codes are optimized for α = 0.1.

Figure 1 Left depicts the evolution of the time series generated by two groups of neurons, each one composed of 500 neurons, for α = 0.1, 40 stimuli, and a transition interval of 400. Each series is clearly divided in two epochs, the first one corresponding to the time window [0, 5000], in which no relevant event appears, and a second window [5000, 10000] in which neurons respond to rare external stimuli. As previously described, an efficient code requires important stimuli to be codified with short symbols, which, in turns, are associated with high spike densities. This effect is clearly shown in Figure 1 Left, where the proportion of spiking neurons after time 5000 is roughly increased by 0.05.

As neural codes are independently generated for the 1000 neurons considered, with different probability distributions, and external stimuli are also triggered in an independent way, no synchronization is expected between both time series. Indeed, if one computes the Pearson's correlation coefficient between both series within the time window [0, 5000], the result is in the order of 10^−4^. Nonetheless, an interesting result is obtained when the correlation is calculated by means of a sliding window; in other words, a time-varying correlation is obtained, whose value at time *t* represents the dynamics of both neural groups in the interval [*t* − 200, *t* + 200]. Intuitively, when analyzing the series near time 5000, both series share the same trend, i.e., an upward dynamics, thus leading to a positive synchronization. Such effect is shown in Figure 1 Left, black line and right scale: around time 5000 the Pearson's correlation coefficient jumps to 0.6.

To confirm this result, Figure 1 Right reports the average synchronization level obtained in 100 realizations of the previously described process, as obtained by 4 commonly used metrics for the assessment of synchronization in brain activity:

- Correlation: Pearson's linear correlation between the two time series.
- Granger causality: following the original definition in Wiener (1956), a time series is said to cause a second one if one can improve the prediction of the evolution of the latter by incorporating information about the past dynamics of the former. Such relationship is tested by means of bivariate autoregressive models (AR). The value here reported is the value of 1 − α^*^, α^*^ being the critical level of significance for which the first time series can be considered causal to the second one.
- Mutual information: assesses the quantity of information, measured in *bits*, that two time series share. In other words, it measures how much knowing one of these time series reduces uncertainty about the other.
- Synchronization Likelihood: arguably one of the most popular index for assessing the presence of generalized synchronization, returns a normalized estimate of the dynamical interdependencies between two or more time series (Stam and Van Dijk, 2002). It relies on the detection of simultaneously occurring patterns, even when they are different in the two signals.

As can be seen in Figure 1 Right, all four metrics present a peak around time 5000, indicating that they all detect this spurious synchronization between the two groups of neurons.

This spurious synchronization is caused by the optimization of the neural code, in which the length of important events is minimized, thus increasing the proportion of spiking neurons when rare events are presented to the system.

The example proposed in Figure 1 Left is not very ecological as the set of events presented in the two halfs of the considered period only included frequent ([0, 5000]) and infrequent ([5000, 10000]) events. Figure 1 Center presents a more realistic example, in which the probability of finding rare events is continuously varied between two intermediate values. The resulting time series (gray and light red lines) are highly noisy, while it is still possible to detect some trends. The black solid line represents the evolution of the Pearson's correlation coefficient calculated over a sliding window of size 400. Even in this noisy configuration, it is possible to detect regions in which the correlation between the two time series is strongly increased - similar results were obtained with the three other considered metrics.

## 3. Discussion

In conclusion, we showed that synchronization can appear when the response of two groups of binary neurons is modulated by the simultaneous appearance of uncommon stimuli, even if both groups do not share information and are not performing a common computation. This is due to the way neural codes are constructed, i.e., to the preference of short symbols, with high spiking rates, representing uncommon events. The present toy model is not intended to mirror actual neural functioning, but rather to draw attention to a possible source of spurious synchronization occurring at the system level of description of neural activity typical of standard neuroimaging techniques. In particular, our results show that even a measure such as the Granger causality can be fooled into signaling causal relationships in the presence of mere coincidences corresponding to no underlying computation. This confirms that claims of causality from (multiple) bivariate time series should always be taken with caution (Pereda et al., 2005), as true causality can only be assessed if the set of two time series contains all possible relevant information and sources of activities for the problem (Granger, 1980), a condition that a neurophysiological experiment can only rarely comply with. Finally, it is important to remark that our model's main suggestion that some of the correlations one would observe in neural activity would not correspond to genuine computation holds true even for resting brain activity, which is operationally defined by the absence of exogenous stimulation. This is explained by the fact that resting brain activity is characterized by unobservable, endogenous activity stemming from numerous simultaneous sources rendering spurious coincidences a plausible occurrence.
